# Arboviruses in Mammals in the Neotropics: A Systematic Review to Strengthen Epidemiological Monitoring Strategies and Conservation Medicine

**DOI:** 10.3390/v15020417

**Published:** 2023-02-01

**Authors:** Cinthya García-Romero, Gabriel Alberto Carrillo Bilbao, Juan-Carlos Navarro, Sarah Martin-Solano, Claude Saegerman

**Affiliations:** 1 Maestría en Biodiversidad y Cambio Climático, Facultad de Ciencias del Medio Ambiente, Universidad Tecnológica Indoamérica, Quito 170521, Ecuador; 2 Instituto de Investigación en Zoonosis (CIZ), Universidad Central del Ecuador, Quito 170521, Ecuador; 3 Research Unit of Epidemiology and Risk Analysis Applied to Veterinary Sciences (UREAR-ULiege), Fundamental and Applied Research for Animal and Health (FARAH) Center, Department of Infections and Parasitic Diseases, Faculty of Veterinary Medicine, University of Liège, B-4000 Liège, Belgium; 4 Facultad de Filosofía, Letras y Ciencias de la Educación, Universidad Central del Ecuador, Quito 170521, Ecuador; 5 Grupo de Investigación en Enfermedades Emergentes, Ecoepidemiología y Biodiversidad, Facultad de Ciencias de la Salud, Universidad Internacional SEK, Quito 170521, Ecuador; 6 Grupo de Investigación en Sanidad Animal y Humana (GISAH), Carrera Ingeniería en Biotecnología, Departamento de Ciencias de la Vida y la Agricultura, Universidad de las Fuerzas Armadas—ESPE, P.O. Box 171-5-231B, Sangolquí 171103, Ecuador

**Keywords:** Latin America, non-human primates, systematic review, arboviruses, Venezuelan equine encephalitis (VEEV), Saint Louis encephalitis (SLEV), West Nile virus (WNV)

## Abstract

Arthropod-borne viruses (arboviruses) are a diverse group of ribonucleic acid (RNA) viruses, with the exception of African swine fever virus, that are transmitted by hematophagous arthropods to a vertebrate host. They are the important cause of many diseases due to their ability to spread in different environments and their diversity of vectors. Currently, there is no information on the geographical distribution of the diseases because the routes of transmission and the mammals (wild or domestic) that act as potential hosts are poorly documented or unknown. We conducted a systematic review from 1967 to 2021 to identify the diversity of arboviruses, the areas, and taxonomic groups that have been monitored, the prevalence of positive records, and the associated risk factors. We identified forty-three arboviruses in nine mammalian orders distributed in eleven countries. In Brazil, the order primates harbor the highest number of arbovirus records. The three most recorded arboviruses were Venezuelan equine encephalitis, Saint Louis encephalitis and West Nile virus. Serum is the most used sample to obtain arbovirus records. Deforestation is identified as the main risk factor for arbovirus transmission between different species and environments (an odds ratio of 1.46 with a 95% confidence interval: 1.34–1.59). The results show an increase in the sampling effort over the years in the neotropical region. Despite the importance of arboviruses for public health, little is known about the interaction of arboviruses, their hosts, and vectors, as some countries and mammalian orders have not yet been monitored. Long-term and constant monitoring allows focusing research on the analysis of the interrelationships and characteristics of each component animal, human, and their environment to understand the dynamics of the diseases and guide epidemiological surveillance and vector control programs. The biodiversity of the Neotropics should be considered to support epidemiological monitoring strategies.

## 1. Introduction

Tropical forests harbor much of the world’s tree diversity [1,2] and more than 1617 recognized mammal species [3,4]. Mammals are important ecological components for nutrient distribution [5,6], seed dispersal [7,8], and interactive connectors between animal species and habitats [9,10,11].

The Neotropics region includes much of Latin America, from Mexico to Argentina [12], and is probably the area that harbors the greatest biodiversity on a global scale [13]. Among the species currently recognized in biogeographic regions, the Neotropics harbor the largest number of mammal species (1617 species), followed by the Afrotropics (1572 species), the Palearctic (1162 species), and Australasia–Oceania (527 species) [3].

Within the world list of seventeen megadiverse countries, six of them (Mexico, Venezuela, Colombia, Ecuador, Peru, and Brazil) are part of the Neotropics [14]. In addition, this region includes areas of high “hotspot” diversity and nine areas of endemism or species richness [15,16] that are highly threatened [17].

In the case of mammals, 60% of threatened species are located in hotspots [18], with the highest percentage of declining species concentrated in the Neotropics [19]. For the Neotropics and its diversity, climate change and change use of land are some of the greatest threats [16,20,21], as it influences the occurrence of infectious diseases in various types of the environment [22], as well as the distribution patterns of hosts, and their pathogens and vectors [23,24,25].

Arboviruses are a group of viruses that are transmitted from infected hosts to susceptible hosts by hematophagous arthropod vectors [26,27]. With the exception of African swine fever virus DNA virus, arboviruses are RNA viruses [28] that belong to one of eight families: *Togaviridae* (genus *Alphavirus*), *Flaviviridae* (genus *Flavivirus*), *Peribunyaviridae*, such as genus *Orthobunyavirus*, *Nairoviridae*, such as genus *Orthonairovirus*, *Phenuiviridae*, such as genus *Phlebovirus*, *Reoviridae* genus *Orbivirus*, *Rhabdoviridae* genus *Vesiculovirus*, and *Orthomyxoviridae* genus *Thogotovirus* [29,30,31]. Approximately 500 arboviruses are known, of which 100 can cause diseases in humans and 40 in domestic animals [32,33].

The success of virus transmission is determined by the interaction between the virus, the vector, the host, and their environment [34]. Establishing the orders of mammals that act as reservoirs is complicated [35], as arboviruses have a wide range of mammalian hosts that could act as potential reservoirs [36] and amplifiers in wild and domestic environments [37].

Vectors serve as reservoirs, amplifiers, and carriers in wild and domestic settings [38]. Hematophagous arthropods are considered active vectors of arboviruses when they are able to ingest a given pathogen by feeding on an infected vertebrate host, followed by the replication of the pathogen in the vector and subsequent transmission to a new vertebrate host [39].

Infection by an arthropod vector is often necessary to maintain the transmission cycle of arboviruses [40]. In tropical and subtropical regions, there is a great diversity and abundance of potential vectors [41]. Therefore, the presence of a diversity of arthropod vectors poses an impact on the health of humans, wildlife, and domestic animal components. Generally, arthropod vectors are insects such as *Aedes* spp. and *Culex* spp. mosquitoes [42,43], sandflies [44,45], and ticks, mainly from the families *Ixodidae* and *Argasidae* [46,47,48], which circulate with great ease allowing the spread of diseases.

There are several factors that favor the increase in the incidence of arbovirus-caused diseases; for example, the availability of hematophagous vectors [49], urbanization [50,51], global transportation systems [52], deforestation in areas with high levels of biodiversity [53,54,55,56,57], and irrigation systems [58].

Although studies focused on the dynamics of arthropod-borne infectious diseases continue to advance [59], they still pose a conservation risk, mainly in tropical regions, as their prevention and control depend largely on the surveillance of arthropod vectors [60,61,62]. The great diversity of mammals in the wild, and especially in orders that have a more direct association with human activities, entails special attention because they can act as hosts or reservoirs of arboviruses [63,64].

Some groups of mammals are considered good reservoir hosts and/or amplifying reservoirs; for example, the orders Rodentia and Chiroptera are the most numerous with worldwide distribution and present a variety of locomotor adaptations that allow them to have a great capacity to adapt to new habitats [65]. Similarly, the order Didelphimorphia presents a wide distribution in the Neotropics in almost all habitat types [66]. These orders involve special attention due to their biological characteristics, as they act as wild reservoirs of arboviruses [36] that affect humans and domestic animals [67,68].

The emergence and spread of emerging infectious diseases are associated with the way humans interact with animal species and the environment [69]. The importance of detecting the zoonotic spread of a vertebrate animal, beyond being a natural reservoir, is to understand a complex process that requires the intervention of environmental, pathogen, and host factors [70,71]. Habitat loss increases biodiversity loss [69]. Better-preserved habitats reduce spillover events, which is known as the dilution effect [72]. Higher diversity often leads to a lower prevalence of infection in hosts [73].

This research proposes to contribute to public health and zoonosis or re/emerging diseases prevention considering that the niche, vector dynamics, hosts, and viruses have been modified in the last decades. In certain habitats, accelerated population growth, quality of life, and sanitary conditions could favor the spread of arboviruses in various mammalian hosts [33,74], posing a global public health problem [47,75].

In order to achieve a better understanding of the ecology of diseases, it is necessary to detect, prevent and control them, and to approach them from a conservation medicine approach given the connection between wildlife and domestic animals, the ecosystem, and humans [76]. This approach will serve as a tool for the understanding, prevention, and management of health problems derived from environmental change [77] in one of the most biodiverse areas of the planet.

This systematic review will allow the identification of studies focused on mammalian arboviruses in the Neotropics excluding the human primate group. The analyses will show the diversity and geographic records of arboviruses, as well as the taxonomic groups of mammals that are most monitored and those that have an information gap. The information obtained on the association of wild and/or domestic mammals as potential hosts and reservoirs of arboviruses is intended to support epidemiological surveillance tasks with a focus on conservation medicine and/or the One Health approach [78,79] to achieve the integration of knowledge and apply it in favor of biodiversity.

## 2. Methods

The literature review of mammalian arbovirus records in the Neotropics was conducted between 1 June and 31 July 2021 under the Preferred Reporting Items for Systematic Reviews and Meta-Analyses PRISMA 2020 Checklist reporting guide and the PRISMA 2020 flowchart [80,81] that describe the process for literature exploration and justification for the selection of each investigation [82,83].

For the search of indexed articles, we used Google Scholar and PubMed databases by combining the following keywords and Boolean operators: Mammals AND arbovirus AND Neotropical countries AND Argentina OR Belize OR Bolivia OR Brazil OR Chile OR Colombia OR Costa Rica OR Ecuador OR El Salvador OR Guatemala OR Guyana OR French Guyana OR Honduras OR Mexico OR Nicaragua OR Panama OR Paraguay OR Peru OR Suriname OR Uruguay OR Venezuela, including all publications without distinction of the year of publication. Additionally, exclusion criteria included (1) a language other than Spanish, Portuguese, and English, (2) a focus on humans, vaccines, clinical cases, and laboratories, (3) duplicates between the two predetermined databases, (4) lack of information regarding the locality of registration, (5) not including the mammalian class, (6) books with extensive and generalized content, and (7) bibliographic reviews.

All the information obtained from the selected articles was organized in a database to determine the sampling effort, i.e., the number of publications as surrogate data, arbovirus records in neotropical countries (number of samples and prevalence), including orders of mammals (wild and domestic) most monitored, laboratory techniques for sample processing used in arbovirus detection, type of habitat (primary forest, secondary forest, intervened area, not specified, populated zone, and captive), and state in which the individual was found at the time of sampling.

Data processing was carried out using SPSS version 20 [84] statistical software. The objective was to identify if arbovirus richness is similar between countries or between mammal genera. Arbovirus richness is the number of arbovirus species found in this study. One positive record was considered each time an article recorded one positive order for one arbovirus; e.g., if one article recorded three arboviruses in one order, three positive records are considered.

Additionally, we determinate the principal orders with arbovirus records in different environments (primary forest, secondary forest, intervened area, not specified, populated zone, and captive) and we provide relevant data to be used to support arbovirus management and prevention protocols in order to contribute to the knowledge of arbovirus of public health concern in the region.

This quantitative tool allowed the statistical interpretation of the results presented in each of the independent studies selected within the systematic review process [85].

We performed a negative binomial regression analysis to determine if there is an increase in the monitoring effort number of publications over time [86]. We used the Kruskal–Wallis equality-of-populations rank test to determine if arbovirus richness is similar between countries or between mammal genera [87,88].

The prevalence values for each publication were calculated as follows:

(1) $$\frac{\#\;\mathrm{positive}\;\mathrm{records}\;}{\#\;\mathrm{total}\;\mathrm{samples}}*100$$

Based on these results, we structured the table in Section 3.1 according to each arbovirus identified, in which the fourth column “n” and the fifth column “prevalence %” contain the minimum and maximum values obtained as a result of the calculation presented.

For the representation of the positive records of arboviruses in mammals of the Neotropics, we generated several maps to identify the areas in each country where the presence of arboviruses was recorded and the orders of mammals monitored in the Neotropics. The data of the geographic points of the sampling sites with positive records for arboviruses were entered into the ArcGIS 10.5 platform [89]. According to the specifications of each investigation, we used coordinates given in the original paper (n = 21) and for the other, we georeferenced based on the locality names (n = 23).

For the graphical representation of viruses recorded in mammalian orders according to habitat, we used GEPHI 0.9.2 software ([90] in which one positive record was considered each time an article recorded one positive order for one arbovirus; e.g., if one article recorded three arboviruses in one order, three positive records are considered).

To determine whether arbovirus records are shared among the countries of the Neotropics, the Jaccard and Sorensen similarity indices were calculated to estimate the compositional similarity of arboviruses among the orders and countries of the Neotropics based on the presence/absence data of positive records identified, using the following formulas [91,92,93]:

(2) $$JI=\frac{A}{A+B+C}$$

(3) $$SI=\frac{2A}{2A+B+C}$$

where *JI* = the Jaccard index; *SI* = the Sorensen index; *A* = unique species per site one; *B* = unique species per site two; and *C* = the number of species in common between two sites.

The two indexes were compared using Pearson’s correlation coefficient.

In addition, a Spearman correlation was applied between the two indices for the number of arboviruses per country, as well as for the number of arboviruses per order.

This analysis made it possible to identify the countries and orders that are most closely related due to various factors such as: host mammal distribution, migrations, trade, and even health policies [94].

The World Wide Fund for Nature has compiled and analyzed in the shape file the global data on significantly increasing deforestation and degradation trends [95], which were used for visualization purposes [96]. We added the positive arbovirus records for the Neotropics identified in the literature review. The characteristics of the habitat described in the articles, either in front of deforestation or with vegetation cover, were taken into consideration.

Finally, based on the analysis of the publications obtained as a result of the literature review, the characteristics, conditions, or behaviors that increase the likelihood of encountering the top-3 arboviruses, the Saint Louis encephalitis virus (SLEV), the Venezuelan equine encephalitis virus (VEEV), and the West Nile virus (WNV) in the Neotropics were identified [97]. The parameters considered include factors such as deforestation, habitat use change, human and animal migration, climate change, behavioral patterns, altered interactions, surveillance, and conservation programs. The purpose was to provide relevant data to be used as a tool to support arbovirus management and prevention protocols, and to contribute to the knowledge of arbovirus diversity of public health concerns in the region. We identified the odds ratio of a mammal being exposed to an arbovirus according to the vegetation cover and deforested fronts, as well as the positive and negative records of arbovirus identified in the nine orders of mammals.

## 3. Results

### 3.1. Arboviruses Richness

**Figure 1:**
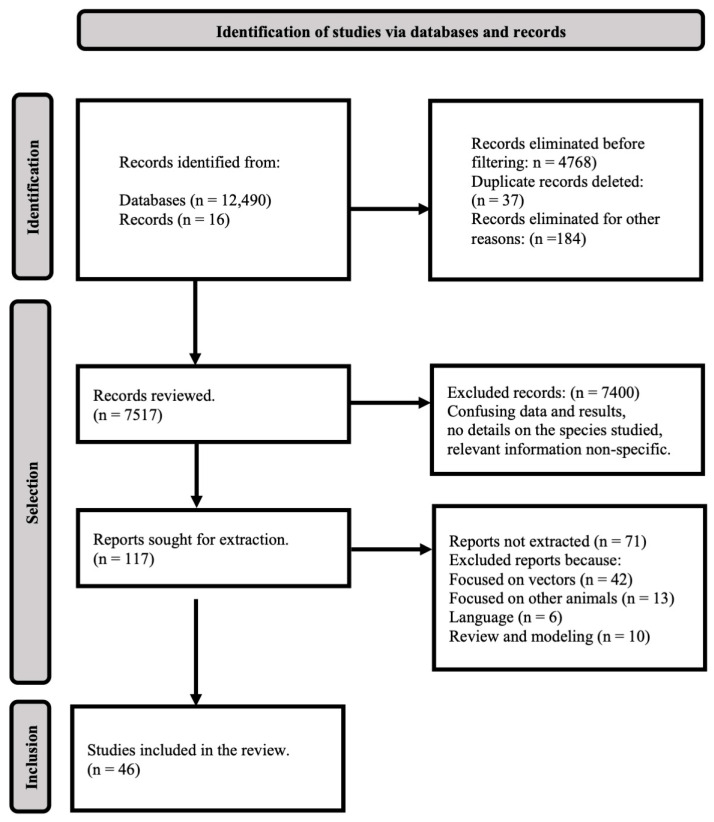
Flow chart modified from (PRISMA 2009) describing the literature search and study selection.

The systematic review was conducted based on PRISMA guidelines (Figure 1) allowed us to quantify the richness of arboviruses (n = 43) present in nine mammalian orders in the Neotropics (Table 1). In the Neotropics region, sampling efforts (n = 46 citations) in the detection of arboviruses in mammals have focused on the following countries: Brazil (n = 15), Argentina (n = 6), Costa Rica (n = 5), Mexico (n = 5), Venezuela (n = 5), Colombia (n = 4), French Guiana (n = 3), Panama (n = 2), Guatemala (n = 1), Paraguay (n = 1), and Uruguay (n = 1). There are several areas harboring the same types of arboviruses, as evidenced in (Table 1). The country with the highest number of arboviruses is Brazil (n = 27). However, no significant differences were found between the arbovirus richness of the countries (Kruskal–Wallis equality-of-populations rank test; Chi2 = 13.474, *p* > 0.05).

### 3.2. Prevalence and Detection Methodology

As for the sample size in the studies obtained, it ranges from one single case (case studies) to 2214 individuals. These results show different prevalences among the arbovirus-positive records ranging from 1–100% but, for a small sample size, the 95% confidence interval of the prevalence of arboviruses is wide, so it induced uncertainty.

For the identification of arbovirus-positive records, the most common sample type tested was blood serum (46.67%). Indeed, no study was recorded that performed tests in which animals were not trapped or disturbed. The most used laboratory techniques were hemagglutination inhibition (HI), with 40%, and a plaque reduction neutralization test (PRNT), with 40%, and a real-time polymerase chain reaction qPCR, with 33.33%. A small percentage of studies applied techniques such as a polymerase chain reaction (PCR), with 8.89%, an enzyme-linked immunosorbent assay (ELISA), with 6.67%, and a tissue-based study of histopathology, with 2.22%.

The analysis of the similarity between countries showed that according to the Sorensen and Jaccard indexes, the groups of countries that share more similarity between arboviruses discovered are Colombia and Panama (Sorensen index = 80.00% and Jaccard index = 66.67%), Guatemala and Mexico (Sorensen index = 58.82% and Jaccard index = 41.67%), Guatemala and Panama (Sorensen index = 50% and Jaccard index = 33.33%), and Mexico and Panama (Sorensen index = 56% and Jaccard index = 38.89%) (Table 2 and Table 3). In addition, the relationship between the two indexes was very high (Spearman’s correlation coefficient = 0.985, with a *p*-value < 0.0001).

The systematic review identified the richness of arboviruses (n = 43) present in nine mammalian orders: non-human primates (n = 20), Perissodactyla (n = 17), Rodentia (n = 18), Artiodactyla (n = 12), Pilosa (n = 11), Chiroptera (n = 8), Didelphimorphia (n = 6), Carnivora (n = 2), and Lagomorpha (n = 2). There are several mammalian orders harboring the same species of arboviruses, as evidenced in (Table A1).

The similarity analysis for mammalian orders evidenced that according to the Sorensen and Jaccard indexes, the orders that share more arboviruses are Chiroptera and Didelphimorphia (Sorensen index = 61.54% and Jaccard index = 44.44%) (Table 4 and Table 5).

**Figure 2:**
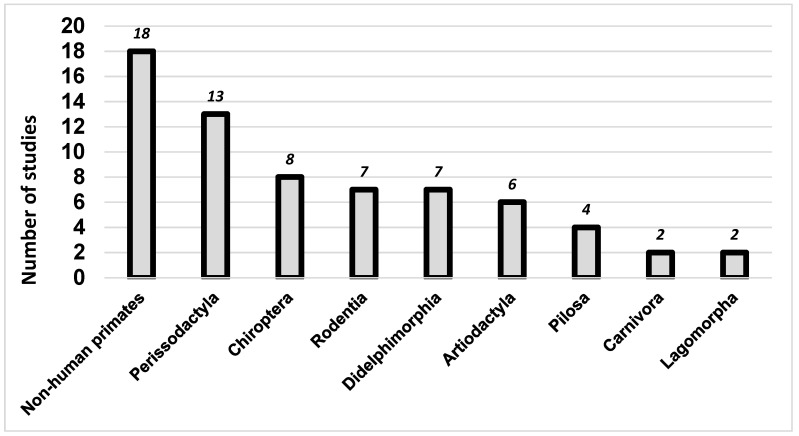
Arbovirus sampling effort in the Neotropics by order of mammal decreasing order.

The results of the literature review identified that monitoring efforts have focused on nine taxonomic orders: mon-human primates (n = 18), Perissodactyla (n = 13), Chiroptera (n = 8), Rodentia (n = 7), Didelphimorphia (n = 7), Artiodactyla (n = 6), Pilosa (n = 4), Carnivora (n = 2), and Lagomorpha (n = 2) (Table A2 and Figure 2).

**Figure 3:**
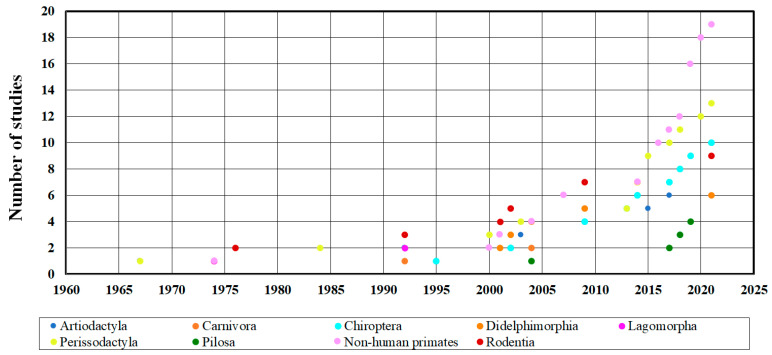
Sampling effort for arboviruses in the different orders of mammals in the Neotropics during the period 1967–2021 (n = 46).

Sampling efforts were conducted during the period 1967–2021. From the periods 2000–2005 and 2015–2021, there is an increase in research focused on arbovirus detection. It is evident that the sampling effort has increased over the years (negative binomial regression; *p* < 0.001) (Figure 3).

**Figure 4:**
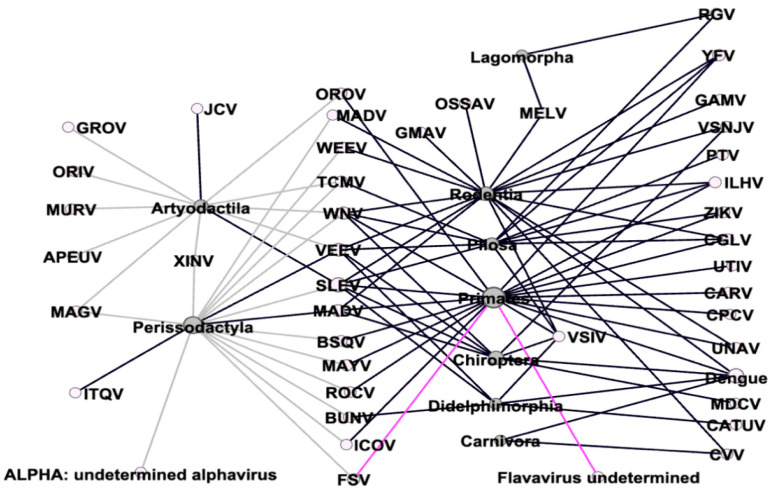
Arbovirus host network in mammals in the Neotropics. Legend: grey link: eomestic environment, black link: wildlife environment, pink link: zoological settings, grey circles: mammal orders, white circles: arbovirus. Note: Apeu virus—APEUV; Bunyamwera virus—BUNV; Bussuquara virus—BSQV; Cache Valley virus—CVV; Cacipacore virus—CPCV; Caraparu virus—CARV; Catu virus—CATUV; Changuinola virus—CGLV; Dengue virus; Madariaga virus—MADV; Western equine encephalitis virus—WEEV; Flavavirus undetermined; Fort Sherman virus—FSV; Gamboa virus—GAMV; Guama virus—GMAV; Guaroa virus—GROV; Icoaraci virus—ICOV; Ilheus virus—ILHV; Itaqui virus—ITQV; Jamestown Canyon virus—JCV; Madrid virus—MADV; Maguari virus—MAGV; Mayaro virus—MAYV; Melao virus—MELV; Mojui dos Campos virus—MDCV; Murutucu virus—MURV; Oriboca virus—ORIV; Oropouche virus—OROV; Ossa virus—OSSAV; Punta Toro virus—PTV; Rocio virus—ROCV; Sant Louis encefalitis virus—SLEV; Tacaiuma virus—TCMV; UNA virus—UNAV; Utinga virus—UTIV; Venezuelan equine encephalitis virus—VEEV; Vesicular stomatitis, Indiana serotype virus—VSIV; Vesicular stomatitis virus, New Jersey serotype—VSNJV; West Nile virus—WNV; Xingu virus—XINV; yellow fever—YFV; Zika—ZIKV; Rio Grande—RGV.

The orders non-human Primates and Rodentia have the highest arbovirus richness (n = 20 and n = 18, respectively). However, no significant difference was found between orders (Kruskal–Wallis equality-of-populations rank test = 14.54, *p* > 0.05). The orders with the highest number of arbovirus-positive records are Primates (n= 54) and Perissodactyla (n = 45) (Figure 4). Wildlife habitat conditions influence arbovirus richness in non-human primates while the domestic environment influences more in the order Perissodactyla.

**Figure 5:**
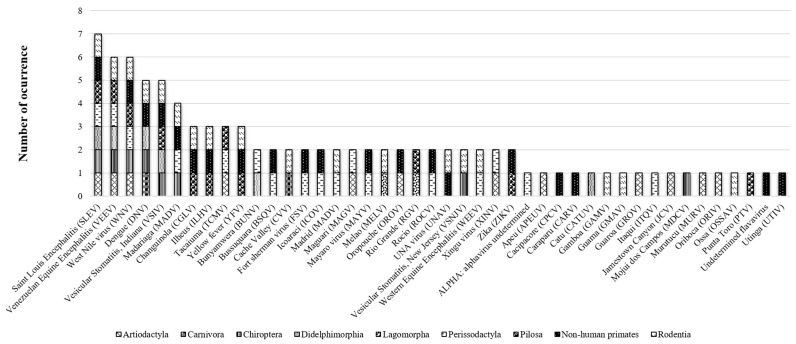
Arbovirus records by mammalian order in the Neotropics.

The arboviruses recorded in most mammalian orders in the Neotropics are the Saint Louis encephalitis virus (SLEV) (n = 7), the Venezuelan equine encephalitis virus (VEEV) (n = 6), and the West Nile virus (WNV) (n = 6) (Figure 5).

**Figure 6:**
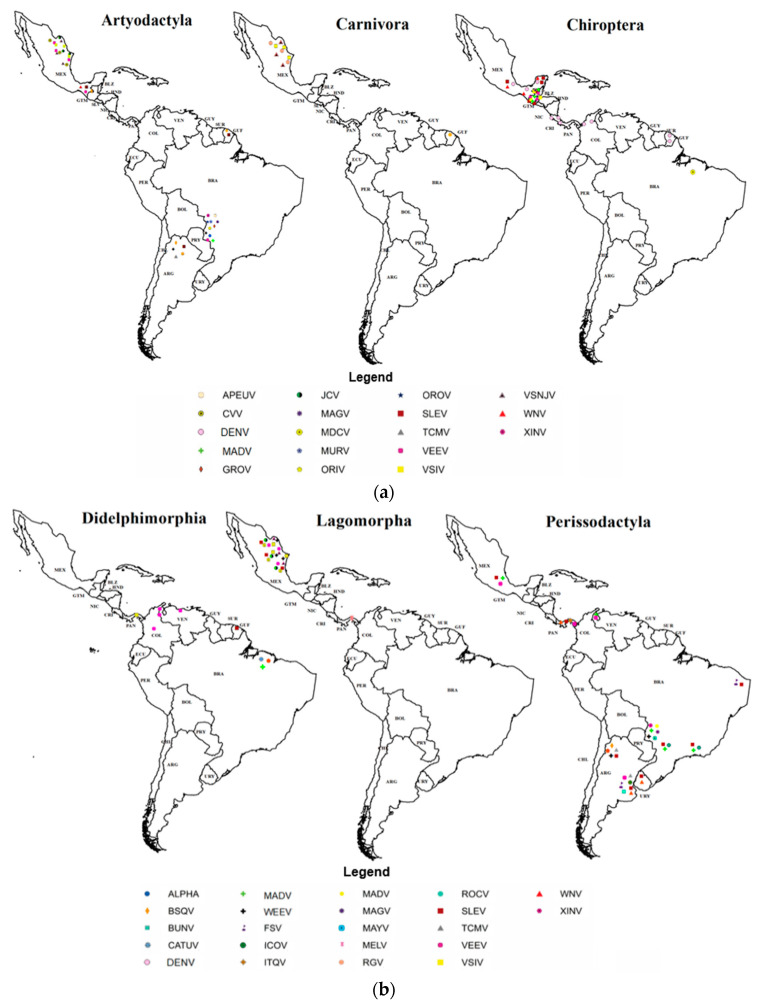

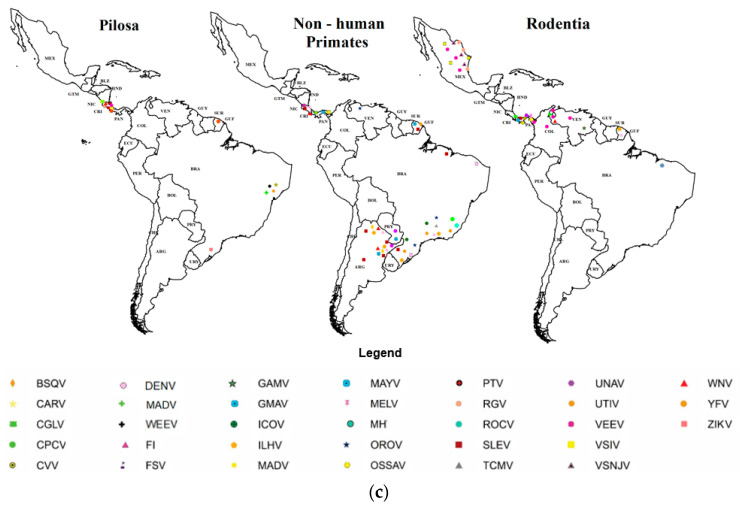
(**a**) Geographical location of arboviruses recorded by mammalian order in the Neotropics. (**b**) Geographical location of arboviruses recorded by mammalian order in the Neotropics. (**c**) Geographical location of arboviruses recorded by mammalian order in the Neotropics. Note: The concordance between the abbreviation of viruses and their full names is derivated from Table 1.

The map (Figure 6) shows the areas reported with the presence of arboviruses according to the orders of mammals monitored and the types of arboviruses identified for the Neotropics. The country with the highest number of positive orders for arboviruses was Brazil, with 27 arboviruses.

### 3.3. Habitat Types with Positive Orders and Sampling Conditions According to Order

**Figure 7:**
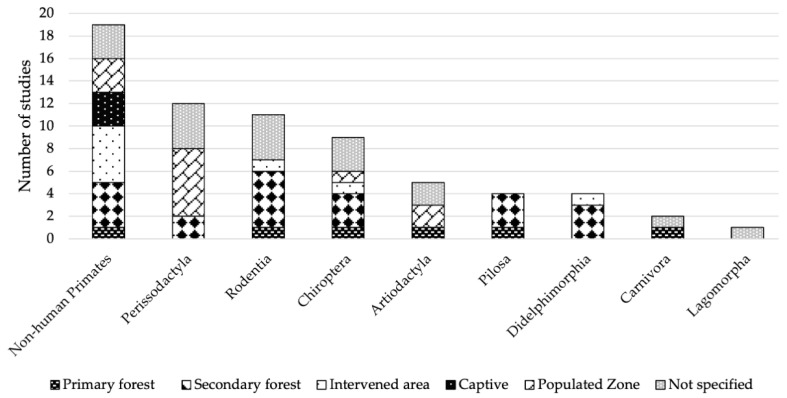
Habitat types with positive records of arboviruses in mammalian orders in the Neotropics.

**Figure 8:**
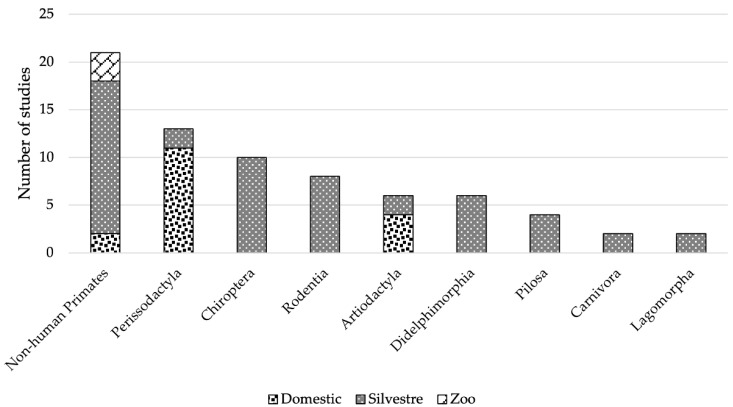
Sampling conditions by mammalian order in the Neotropics.

It was possible to identify that arbovirus records were found in a wild range of sampling habitat types, i.e., primary forest, secondary forest, disturbed areas, captivity, and even in populated areas (Figure 7). In addition, all mammalian orders that were identified as hosts were found in the wild. However, there were also records both in captive conditions and in the domestic environment (Figure 8).

### 3.4. Risk Factors

**Figure 9:**
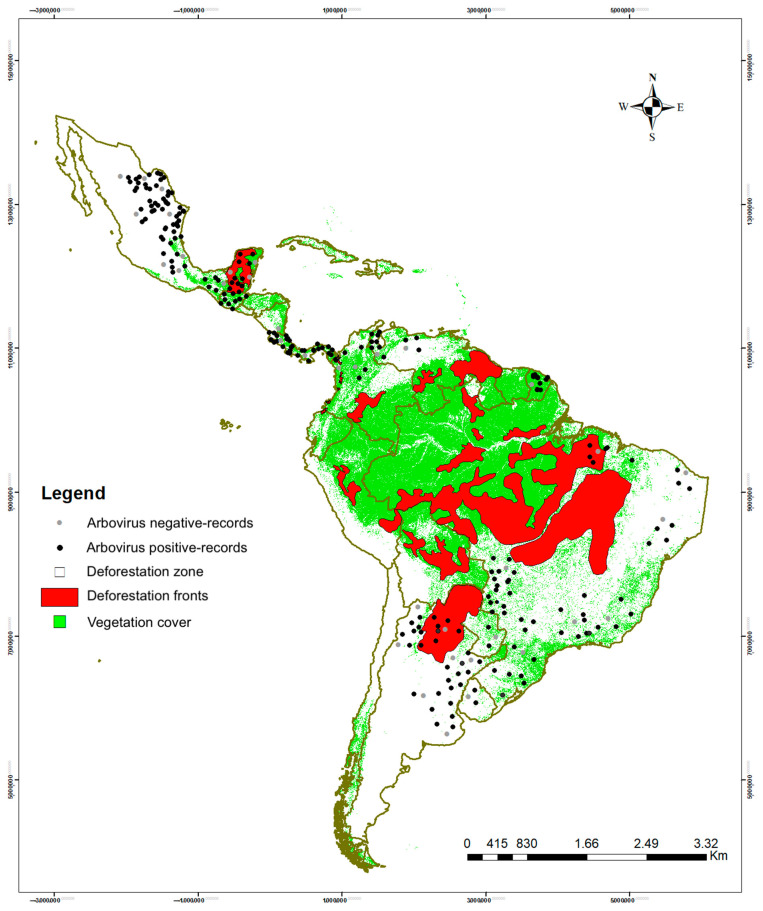
Representation of arbovirus positive and negative records, vegetation cover, and deforested fronts in the Neotropics.

In the Neotropical region, vegetation cover and deforested fronts were identified, as well as the arbovirus positive and negative records that were identified in the nine orders of mammals. The map obtained showed that the arbovirus-positive and negative records for each publication analyzed were mostly found outside the areas with vegetation cover and others were recorded within deforested fronts (Figure 9 and Table A2). The risk factor map includes positive and negative records. All but four studies (which had only positive records) had both negative and positive records. No studies were found with only negative records. The odds ratio of a mammal becoming being exposed to an arbovirus is 1.46 higher when its habitat is located in deforested fronts (95% confidence interval: 1.34–1.59) than if its habitat is with vegetation cover (*p* < 0.0001) (Table 6).

## 4. Discussion

### 4.1. Richness of Arboviruses

The results of the literature review reported that monitoring efforts have been concentrated in Argentina, Brazil, Mexico, and Panama, which may be associated with the diversity of arboviruses and vectors they harbor [142,143]. These countries share the occurrence of three arboviruses, i.e., VEEV, SLEV, and WNV. SLEV and VEEV are neotropical viruses, and WNV is introduced from Africa. They are viruses with complex transmission cycles, a variety of hosts, and a variety of vectors. In addition, in the Neotropics, they are distributed in rural areas, jungle areas, and a few cities on the periphery. SLEV and WNV are Flaviviruses and in humans they exist as cross-protections with the antibodies of the population against dengue and yellow fever, which is why they do not occur in the Neotropics. Dengue and Chikungunya are introduced from Africa and transmitted by the vector *Aedes aegypti*; they are part of an exact transmission cycle in urban areas in the Neotropics [110,144].

Among the countries with arbovirus records are Guatemala, Costa Rica, French Guiana, Paraguay, and Uruguay. In most cases, the pathways for the introduction of arboviruses into new regions are unknown [145]. However, the emergence and spread of arboviruses rapidly and geographically may be due to the growth of global transportation systems [146,147,148,149,150,151] and the adaptation of humans and arthropods due to increasing urbanization [52]. The countries that obtained the highest number of arbovirus similarity are geographically close, such as Colombia and Panama, which share the biogeographic region of El Choco, and Mexico and Guatemala with shared ecosystems, thus creating the possibility of harboring similar arbovirus species in the same ecozone [152,153].

Recently, arbovirus diseases have been reported with increased frequency worldwide [154]. In the Neotropics, Brazil is characterized by encompassing a large land area covered by tropical forests and densely populated areas [155,156]. These characteristics are closely linked to the strengthening of research efforts according to the number of resources invested for research and development in arbovirus research areas [157]. Similarly, surveillance preferences for specific species and the ease of sampling sites favor the detection of arbovirus prevalence due to the high rates of the infected population [158]. These conditions coincide with the results obtained from the systematic review carried out, which reflect that Brazil is the country with the highest arbovirus richness in the region (n = 27). Due to the high biodiversity of environments and components, a large number of arboviruses have been isolated in Brazil [67,134,135], especially those involved in human diseases such as the Western equine encephalitis virus [98], Saint Louis encephalitis virus [125], Mucambo virus [159], Guaroa virus [99], Tacaiuma virus [102], and Guama virus [106].

The ability of transmission vectors to spread is a determining factor for arbovirus outbreaks worldwide. A previous systematic review of arboviruses in Western Europe reflected that current outbreaks are due to the spread of *Aedes albopictus* and *Aedes japonicus* [160]. Dengue is the most important emerging arboviral disease globally [161,162] due to the wide variety of ecosystems in which it is found and the ease of its spread. Studies have also focused on other arboviruses [163]. An example of this is the VEEV [164], whose natural and most efficient vector in Latin America [165] is *Culex* sp. *Melanoconion* [166,167]. The VEEV is recorded in human [168], equine, and bovine serology studies [126,169] and wild animals [101,170], so it tends to replicate in livestock animals and results in higher levels of contagion in rural environments [171].

Changes in ecological conditions favor the creation of new habitats for arbovirus vectors [132,172,173] and may cause arthropods to adapt to new mammalian hosts [52,174], leading to the emergence of new pathogens in the domestic environment, which is sometimes the main reservoir [175]. Infection in domestic animals can increase circulation and human exposure in peridomestic habitats [168,176].

In the Neotropics, the SLEV is found in a wide distribution from Mexico to Argentina via the mosquito vector *Culex* sp. [177]. Initially, outbreaks were localized in the United States with high human case fatality rates [178]. In Argentina, these records are attributed to the expansion of agricultural and urban habitats [179]. Based on the records of this study, the occurrence of SLEV infection was evidenced in countries of the region such as Costa Rica [107], Guatemala [120], Mexico [104], and Uruguay [125,177].

West Nile virus has the ability to infect a wide variety of wild and captive mammals in all regions of the world [180]. Historically, WNV has one of the broadest host ranges [181,182,183], and mosquitoes act as vectors, e.g., *Culex* sp. [184] of arboviruses in humans and equines [185]. However, very few studies associated with infection in mammalian hosts have been conducted during the last decades [181]. Due to the wide distribution of WNV and the association of records of increased prevalence in mammals in urban areas [186], there is a possibility of new outbreaks in most continents [187,188,189,190,191].

### 4.2. Prevalence and Detection Methodologies

The variability in the number of individuals monitored in this review may be linked to the objectives of each study, the sampling effort, the order of mammals sampled, the capture techniques [192], the type of sample analysis [193], and the characteristics of the site [194].

Different types of methodologies were used to capture the mammals studied. For rodents and marsupials, Sherman traps were used [113] and mist nets were used to capture bats [109,120]. In relation to NHP, dart immobilization [103,115] and manual capture [107] were used. These techniques were used to obtain blood, serum, or tissue samples from the vertebrate host, which in some cases studies involved the sacrifice of species, such as rodents and marsupials [101,113,121,132].

In the three arboviruses with the highest number of records, both serological virus isolation and molecular techniques were used. This allows a better confirmation of the presence of arboviruses in individuals [101,118,124,141] due to the materials and equipment necessary for the correct execution of the analysis protocols for each sample, either under culture, molecular, or serological techniques. The most applied detection methodologies for arbovirus detection were hemagglutination inhibition by its capacity or facility and plaque reduction neutralization test. The combined use of serological and molecular techniques facilitates the indirect detection of arboviruses. For example, the simultaneous application of techniques, such as ELISA and RT-PCR, allowed the identification of co-infection of dengue with other arboviruses, such as Chikungunya and Zika, respectively [195].

In the last decade with the significant development of new molecular detection technologies in epidemiological surveillance, there are still few studies using PCRs or qPCRs for arbovirus species determination (16/46) [100,101,196]. However, according to Mendoza-Ponce, Corona-Núnez, Galicia, and Kraxner [16] worldwide, qPCR is highly effective for diagnosing arboviruses in humans, even with low viremias. The accuracy of this technique [65] is important in wildlife, as samples cannot always be repeated or individuals have already died. This methodology is sensitive and specific and should be used in wildlife. Phylogenetic studies that would complement detection studies are not widely present [108,113]. Currently, with the COVID-19 pandemic, the importance of having more specific tools, such as sequencing and metagenomics, at hand for proper species detection and determination of outbreak origins were observed [197,198]. Additionally, advances in equipment mean that samples can be taken and analyzed in the field, reducing data loss [199].

The results of the literature review showed that most of the records were identified in secondary forest mammals, which is associated with previous research showing that areas with greater intervention have greater potential for zoonotic diseases, which could act as a potential danger to surrounding communities [55]. For example, in Singapore, it was reported mosquitoes of the genus *Aedes* could be both in the forest and in urban open areas that are highly frequented by people [200]. Similarly, in Brazil, it was reported there were a high abundance of vectors in urban forests and a dominance of vector species according to habitat [172].

The use of non-invasive samples is an opportunity that could facilitate the diagnosis and detection of arboviruses in animals using urine and saliva samples [201,202,203]. Detection using fecal samples is another cost-effective and non-invasive option to monitor wild populations that could be potential reservoirs of arboviruses [204]. The use of this type of sample is effective for arbovirus detection and is an option to avoid stress on monitored species. Similarly, research focused on this sampling protocol could increase the number of orders that currently have not been monitored and contribute to conservation genetics, as well as behavioral ecology and infectious and parasitic diseases [205,206,207].

It is important to note that, although this study reflects the reality of arbovirus monitoring in the neotropics, there is a lack of study of negative records (zero prevalence). Unfortunately, few studies on infectious and parasitic diseases publish negative results [208]. This study confirms what others have already suggested regarding the publication of positive results [198,209]. There are more publications with positive results than negative ones. In this study, we found no publications with completely negative results. This is called publication bias [210]. Negative results are important because they may have an ecological, behavioral, or management explanation [211], which can directly contribute to our knowledge of infectious and parasitic diseases and contribute to epidemiological monitoring plans or policies. Additionally, in some neotropical countries, most research remains unpublish for economic reasons or publication rejections [212], which favors the perception that it is better not to publish negative results. Publication of negative results is highly recommended.

### 4.3. Risk Factors

Climate change implies impacts on human health and vector-borne infectious disease burden [213]. Fluctuating climatic conditions such as precipitation [214], temperature [215], and humidity [216] impact infection rates in tropical regions [217,218] that facilitate arbovirus reproduction and transmission in a wide diversity of habitats [156]. Patterns of vector population distribution, reproduction, and competition [219] can be influenced by temperature [220], precipitation [221], abundance, and the affinity of the vector for a mammalian host species [222]. Variability among hematophagous arthropod species [75], mammalian hosts [223], and the environments or niches they occupy [224] impacts arbovirus transmission dynamics [37,192] and are subject to changes in temperature and precipitation [186,225].

The current co-circulation of three arboviruses, Zika, dengue, and Chikungunya, spreading globally in the Americas suggests the need for more integrative studies and the use of new approaches to identify the cause and risk posed by these combinations [226]. González-Salazar, Stephens, and Sánchez-Cordero [221] even created a model of possible mammalian hosts of Zika for the Neotropical region where seven of the nine species identified were bats. As for Chikungunya, this was not recorded in any order of non-human mammals in the records of the Neotropics, although a study in Brazil carried out serological and molecular analyses of bats without any detection [227]. Based on this background, the question now arises whether Chikungunya and Zika have been found in non-human hosts in America for their maintenance cycle, similar to what occurred with the yellow fever virus and the Mayaro virus, which originated in Africa. Currently, incorrect diagnoses are made based on known symptoms in areas where several arboviruses are circulating simultaneously [228].

On the other hand, in countries in other regions of the world there have already been records of this arbovirus in NHP [229], rats [230], bats [230], and horses [231]. Monitoring and detection should be continued in mammals such as primates, rodents, and bats that are susceptible to infection [232], even without the intervention of a vector [233].

Arbovirus circulation can occur in sylvatic or urban cycles [234]. Thus, altering the balance of natural systems can increase vector abundance, create new reservoirs, or induce arboviruses to adapt to new maintenance cycles [235]. Additionally, the relationship that humans maintain with various species of domestic mammals is considered another factor that favors the reproduction cycles of arboviruses and their vectors [100,236].

### 4.4. Socio-Ecological Aspects

Records of arboviruses in new areas are linked to the distribution and spread patterns of vectors, such as *Aedes aegypti*, from Africa to the Neotropics due to the influence of human trade routes [237]. Social phenomena, such as human migration and species trafficking, are key factors that have favored the spread of arboviruses [60]. Factors that determine vector trajectory conditions are established in various regions [238]. These factors are associated with climate change [239], deforestation [240], uncontrolled growth of urban areas [241], difficulty in accessing clean water sources [242], and population displacement [243]. In the Brazilian Amazon region, there are a greater number of positive records of sylvatic arboviruses associated with deforestation [155], mining [244], road expansion [245], and urbanization with the emergence and/or re-emergence of relevant arboviruses [172].

### 4.5. Ecological Aspects

Environmental factors and environments are determinants in the life cycles of arboviruses as they determine their distribution and dispersal patterns, as well as their transmission to mammalian hosts [40]. The interaction between vectors and mammalian hosts conditions the dynamics and impact of arboviruses in human and domestic animal communities [32].

Arboviruses have a high host-specific association, so changes in range or distribution significantly influence their adaptations in new areas [176]. For example, as shown in Figure 7, NHP is the order identified in the greatest variability of habitats in primary forests, secondary forests, disturbed areas, captivity, and populated areas. Non-human primates are reservoirs for a large number of blood-borne pathogens and ecological factors such as host density, climate change, and activities facilitate the transmission of these pathogens [207].

As a survival mechanism, arboviruses have the ability to develop adaptive mutations when they reach new territories new hosts, vectors, and environments to adapt quickly and improve transmission. Thus, positive records of arboviruses are reflected in variability of sampling conditions, including domestic, wild, and zoo, which can be seen in Figure 8, with the wild condition being the most frequent in all orders. However, in the face of changes in the environment, it is difficult to predict the speed of species response [225,246], so research on the ecology of interactions between arboviruses and mosquito vectors is needed to understand the dynamics of invasion and adaptation in new areas [165].

Ecological mechanisms are an important part of every stage and enzootic potential of the Neotropics, and we must consider within them the exposure of wildlife, the pressures of propagation, the enzootic infection that affects the exposed animals, and the persistence of enzootic transmission [63]. The impact of deforestation on the abundance of vectors, which facilitate arbovirus transmission, influences their movement from wild areas to urban or rural areas [247] where they can adapt to new domestic hosts that maintain direct contact with humans. Figure 9 reflects that the effects of deforestation in forested areas of the Neotropics are more evident over time and increase the contact of the wide variety of vectors with humans, and thus the risk of contagion, dispersal, and epizootics [248,249].

### 4.6. Health Policies

Population growth [250], the expansion of the agricultural frontier [251], the impact of anthropogenic activities [252], and climate change [253] contribute to the spread of arbovirus infections [254]. The 2015 Lancet Commission on Health and Climate Change mentions that the fight against climate change could be the greatest opportunity for global health in the twenty-first century [255].

The levels of epidemiological and entomological surveillance should be potentiated, as well as the joint analysis of the factors that condition the level of vulnerability of a certain area to arboviral diseases [256]. Success in the execution, monitoring, and evaluation of programs focused on the prevention and control of emerging diseases will mitigate the spread of arboviruses in the Neotropics.

At the international level, arthropod vector-borne arboviruses are part of the public health problem that requires cooperation and joint research to establish effective control strategies. All the parameters that are part of the biology of the vectors must be taken into account, as well as the dynamics of reproduction of the arboviruses [257]. Institutions such as the Fundação de Medicina Tropical Dr. Heitor Vieira Dourado FMT-HVD in Brazil work on the detection of arbovirus infections, considering that the country has suffered silent outbreaks due to problems with the identification of arbovirus [156].

The World Organisation for Animal Health (OIE) seeks to promote the importance of animal disease surveillance and communication systems. By generating the necessary knowledge, it will be possible to manage risks, evaluate priorities and generate policies according to the guidelines of each country [258]. Additionally, eradication programs [259,260] consider the social, economic, and even political part of each zone in order to mitigate or control arbovirus infections in the Neotropics.

### 4.7. Actions to Take

The need to strengthen research, surveillance programs, and public policies is indispensable in the Neotropics. The identification of possible regions and habitats with ecological conditions suitable for the circulation of arboviruses and/or specific areas with a high risk of infection is required [261]. In addition, climate change-based modeling associated with infectious diseases supports early warning systems [262].

There should be an increase in the use of non-invasive techniques. The protocols used for sampling in each publication analyzed in the systematic review do not establish non-invasive methodologies, even though several species of mammals that registered positive arboviruses fall within a threat category on the International Union for Conservation of Nature’s (IUCN) red list.

Due to the complexity of transmission and contagion dynamics, as well as the biology of vectors and hosts, it is considered that an integrated solution would allow effective vector control through new technologies, adequate management of space and resources, and control and sanitation policies [263]. For example, in the last decade, the use of geographic information systems (GIS) has become an important tool for the detection, analysis, and prediction of epidemiological patterns that have contributed to the prevention and control plans of diseases caused by arboviruses [264]. In the case of vectors that transmit arboviruses, a biotechnological tool has been generated that is helping to control mosquito vectors by genetically modifying them. It is highly effective, does not harm the environment, is efficient, and mainly has a low production cost [265]. The use of insecticides, on the other hand, has shown that it has created resistance in mosquitoes of the genus *Aedes* [266] that have developed resistance against insecticides. These mosquitoes are transmitters of different arboviruses such as dengue [267], yellow fever [268], Chikungunya [269], and Zika [270].

Long-term monitoring of the interactions of nearby populations is necessary to prevent the spread of arboviruses to uncontaminated locations [271]. It is essential to strengthen epidemiological monitoring in the areas that maintain research initiatives for the control of arboviruses in the Neotropics. Considering that knowledge is the basis for the prevention and control of emerging diseases, in areas that do not yet maintain plans and/or policies of epidemiological monitoring, basic but transcendental measures should be considered, such as training the general population for the elimination of spaces that could serve as potential reservoirs for vectors [272]. Similarly, health education campaigns should be strengthened [273] and encourage research for the early detection of arboviruses, mainly in the areas most likely to be infected [274].

## 5. Conclusions

This study identified the diversity of arboviruses in the Neotropics region, within the list of the 17 countries that make it up. We recorded a greater sampling effort in 11 countries (Argentina, Brazil, Colombia, Costa Rica, Guatemala, French Guiana, Mexico, Panama, Paraguay, Uruguay, and Venezuela). However, there are countries in which no effort has been made, despite the great biodiversity of the area and the wide range of host mammals.

The Venezuelan equine encephalitis virus (VEEV), St. Louis encephalitis virus (SLEV), and the West Nile virus (WNV) share the most mammalian orders. In addition, it was identified that there are orders with greater sampling effort that can be associated with ease in terms of the sampling technique and the objectives of each study. The prevalence of arboviruses (1–100%) varies due to the sample size of each study, as cases range from 1 to 2214 individuals found in various habitats and conditions.

As the present research highlights the record of the Mayaro virus in mammalian hosts and the influence of climate change that facilitates the creation of new environments and the adaptation of vectors, it would be important to focus the monitoring effort on determining if we can consider the Mayaro virus as the next emerging arbovirus given its phylogenetic closeness to the Chikungunya virus [228,275,276]. Furthermore, in the case of Mayaro, due to environmental changes, contact between peri-urban and urban areas is increasingly possible. Finally, experimentally, competition between three vectors (especially *Ae. aegypti* and *Ae. albopictus*, and to a lesser extent *Cx. quinquefasciatus*) has been observed, which means that these vectors may also play a role in the circulation of Mayaro [277,278,279].

Deforestation was observed as an important risk factor in terms of the observed records, as a large number of positive records for arbovirus number of publications are found outside forested areas and within deforested fronts of the Neotropics, which is associated with the fact that disturbed habitats increase the risk of infection [73].

The studies analyzed in the review maintain conventional analysis techniques, such as serology. However, the use of molecular detection tools, e.g., PCR and qPCR, is a priority to have an adequate response in the case of outbreaks. Response tools require monitoring in wild areas to be able to relate outbreaks to origins, as well as the application of techniques that provide more specific information, such as sequencing and metagenomics for the detection of arboviruses.

The orders with the greatest monitoring effort are non-human primates, Perissodactyla, Chiroptera, and Rodentia. In addition, research gaps were identified. Future research should focus on the orders Artiodactyla, Carnivora, Chiroptera, Didelphimorphia, Lagomorpha, Perissodactyla, Pilosa, NHP, and Rodentia, which are important sources of information for disease monitoring. In biodiverse areas, such as Amazonian ecosystems, there are information gaps, so research should focus on the dynamics of emerging diseases and local fauna as a monitoring tool for conservation [245,280].

Interactions between factors such as environment, hosts, and vectors are a potential risk to disease prevalence. Long-term and constant monitoring is required, accompanied by monitoring and sampling techniques focused on methodologies with non-invasive techniques that are cost-effective and provide the same results without altering the dynamics of populations or the health of individuals.

Health education through the generation of educational instruments and constant training for the population is a good option on a smaller scale that could complement the monitoring and prevention initiatives of public policies in each country. Anthropic effects have a direct impact on the factors that determine the trajectory of vectors, the distribution ranges of host mammals, and the distribution of arboviruses in the Neotropics.

## Figures and Tables

**Table 1:** Diversity of arboviruses in mammalian orders in the Neotropics.

Arbovirus	Mammalian Host	Country	Number	Prevalence (%)	Methods	Type of Sample	Reference
ALPHA: alphavirus undetermined	Perissodactyla	Brazil	985	24.26	PRNT	Blood	[98]
Apeu virus (APEUV)	Artyodactyla	Brazil	607	82.2	PRNT	Serum	[99]
Bunyamwera virus (BUNV)	Perissodactyla Didelphimorphia	Argentina Venezuela	2	100	PRC qPCR	Tissue	[100,101]
Bussuquara virus (BSQV)	Perissodactyla Non-human primates	Argentina Brazil Mexico	108 72	77.33 64.81	PRNT	Serum	[102,103,104]
Cache Valley virus (CVV)	Carnívora Rodentia	Mexico Panama	2214 80	1.25 1.26	HI	Blood Serum	[105,106]
Cacipacore virus (CPCV)	Non-human primates	Brazil	139	17.98	HI PRNT	Serum	[102]
Caraparu virus (CARV)	Non-human primates	Brazil	139	17.98	HI PRNT	Serum	[102]
Catu virus (CATUV)	Didelphimorphia	Brazil	14	57.14	HI	Serum	[67]
Changuinola virus (CGLV)	Pilosa Non-human primates Rodentia	Colombia Costa Rica Panama	2214 4109	1.26 79.81	HI	Blood Serum	[106,107]
Dengue virus (DENV)	Carnivora Chiroptera Didelphimorphia Non-human primates Rodentia	Argentina Brazil Colombia Costa Rica French Guiana Mexico	616 16	64.81 0.69	ELISA HI PCR PRNT qPCR	Blood Serum Tissue	[102,103,108,109,110,111,112,113,114,115,116,117,118]
Madariaga virus (MADV)	Chiroptera Perissodactyla Non-human primates Rodentia	Brazil Guatemala Mexico Venezuela	2214 14	93.75 1.26	HI PRNT	Blood Serum Tissue	[67,98,102,104,119,120,121,122]
Western equine encephalitis virus (WEEV)	Perissodactyla Rodentia	Brazil Mexico	985 75	77.33 1.25	HI PRNT	Blood Serum	[98,105]
Saint Louis encephalitis virus (SLEV)	Artiodactyla Chiroptera Didelphimorphia Perissodactyla Pilosa Non-human primates Rodentia	Argentina Brazil Colombia Costa Rica Guatemala French Guiana Mexico Panama Uruguay	2214 1	100 1.25	ELISA HI PRNT qPCR	Blood Serum	[102,103,104,105,106,107,110,111,112,114,116,120,123,124,125,126,127,128,129,130,131]
Venezuelan equine encephalitis virus (VEEV)	Artiodactyla Chiroptera Didelphimorphia Perissodactyla Pilosa Rodentia	Argentina Brazil Colombia Costa Rica Guatemala Mexico Panama Venezuela	985 64	93.75 0.69	HI PRNT qPCR	Blood Serum Tissue	[98,101,104,105,106,107,120,121,122,123,126,129,132,133]
Vesicular stomatitis, Indiana virus (VSIV)	Chiroptera Didelphimorphia Pilosa Non-human primates Rodentia	Colombia Costa Rica Guatemala Mexico Panama	2214 80	79.81 1.25	HI PRNT	Blood Serum	[105,106,107,120]
Vesicular stomatitis, New Jersey virus (VSNJV)	Chiroptera Rodentia	Guatemala Mexico	332 80	26.2 1.25	HI PRNT	Blood Serum	[105,120]
Yellow fever virus (YFV)	Pilosa Non-human primates Rodentia	Brazil French Guiana	574 1	100 8.71	HI PRNT qPCR	Blood Serum Tissue	[102,112,114,128,134]
Undetermined flavavirus	Non-human primates	Costa Rica	86	40.69 44.18	PRNT	Serum	[110,111]
Fort Sherman virus (FSV)	Perissodactyla Non-human primates	Argentina Brazil	222 50	76.8 1.04	HI PRNT qPCR	Blood Serum	[123,135,136]
Gamboa virus (GAMV)	Rodentia	Venezuela	543	5.52	qPCR HI	Blood Tissue	[101]
Guama virus (GMAV)	Rodentia	Colombia Panama	2214	1.26	HI	Serum	[106]
Guaroa virus (GROV)	Artiodactyla	Brazil	607	82.2	PRNT	Serum	[99]
Icoaraci virus (ICOV)	Perissodactyla Non-human primates	Argentina Brazil	222 26	76.8 17.98	HI PRNT qPCR	Blood Serum	[123,124]
Ilheus virus (ILHV)	Pilosa Non-human primates	Argentina Brazil Costa Rica	139 14	79.81 17.98	HI PRNT qPCR	Blood Serum Tissue	[67,102,103,107,124]
Itaqui virus (ITQV)	Perissodactyla	Venezuela	64	93.75	HI	Serum Tissue	[122]
Jamestown Canyon virus (JCV)	Artiodactyla	Mexico	80	1.25	HI	Blood	[105]
Madrid virus (MADV)	Perissodactyla Rodentia	Colombia Panama	2214 194	64.94 1.26	HI	Serum	[106,126]
Maguari virus (MAGV)	Artiodactyla Perissodactyla	Brazil	607	82.2	PRNT	Serum	[99]
Mayaro virus (MAYV)	Perissodactyla Non-human primates	Argentina Brazil French Guiana Panama Paraguay	2214 90	74.44 1.26	HI PRNT	Blood Serum	[98,106,112,137]
Melao virus (MELV)	Lagomorpha Rodentia	Colombia Panama	2214	1.26	HI	Serum	[106]
Mojui dos Campos virus (MDCV)	Chiroptera	Brazil	1	100	HISTOL	Blood	[138]
Murutucu virus (MURV)	Artiodactyla	Brazil	607	82.2	PRNT	Serum	[99]
Oriboca virus (ORIV)	Artiodactyla	Brazil	607	82.2	PRNT	Serum	[99]
Oropouche virus (OROV)	Artiodactyla Non-human primates	Brazil Venezuela	607 1	100 4	HI PCR PRNT	Blood Serum Tissue	[99,128,136,139]
Ossa virus (OSSAV)	Rodentia	Colombia Panama	2214	1.26	HI	Serum	[106]
Punta Toro virus (PTV)	Pilosa	Costa Rica	109	79.81	HI	Blood	[107]
Rio Grande virus (RGV)	Lagomorpha Pilosa	Costa Rica Mexico	109 80	79.81 1.25	HI	Blood	[105,107]
Rocio virus (ROCV)	Perissodactyla Non-human primates	Brazil	753 139	55.11 17.98	ELISA HI PRNT	Serum Tissue	[102,130]
Tacaiuma virus (TCMV)	Artiodactyla Perissodactyla Pilosa	Argentina Brazil	222 75	77.33 17.98	HI PRNT	Blood Serum	[102,123,129]
UNA virus (UNAV)	Non-human primates Rodentia	Argentina Colombia Panama Paraguay	2214 90	74.44 1.26	HI PRNT	Blood Serum	[106,137]
Utinga virus (UTIV)	Non-human primates	Brazil	139	17.98	PRNT	Serum	[102]
West Nile virus (WNV)	Artiodactyla Chiroptera Perissodactyla Pilosa Non-human primates Rodentia	Argentina Costa Rica Mexico Panama Uruguay Venezuela	425 72	79.81 18.57	ELISA HI PRNT	Blood Serum Tissue	[103,104,107,111,115,116,121,123,125,126]
Xingu virus virus (XINV)	Artiodactyla Perissodactyla	Brazil	607	82.2	PRNT	Serum	[99]
Zika virus (ZIKV)	Pilosa Non-human primates	Brazil	132 10	100 6.81	PRNT qPCR	Blood Tissue	[140,141]

Legend: ELISA, enzyme-linked immunoadsorbent assay; HI, hemagglutination inhibition; HISTOL, histology as tissue-based study; PCR, polymerase chain reaction; PRNT, plaque reduction neutralization test; qPCR, quantitative real-time polymerase chain reaction.

**Table 2:** Arbovirus similarity index according to Sorensen’s index among Neotropical countries.

	Argentina	Brazil	Colombia	Costa Rica	Guatemala	French Guiana	Mexico	Panama	Paraguay	Uruguay	Venezuela
**Argentina**											
**Brazil**	45.00										
**Colombia**	36.36	16.67									
**Costa Rica**	47.62	21.05	36.36								
**Guatemala**	25.00	24.24	37.50	40.00							
**French Guiana**	37.50	24.24	25.00	26.67	20.00						
**Mexico**	41.67	30.00	36.36	54.55	58.82	23.53					
**Panama**	50.00	26.32	80.00	43.48	50.00	25.00	56.00				
**Paraguay**	30.77	6.67	15.38	0.00	0.00	28.57	0.00	30.77			
**Uruguay**	30.77	6.67	14.29	33.33	28.57	28.57	28.57	28.57	0.00		
**Venezuela**	35.29	17.65	11.76	25.00	36.36	0.00	33.33	33.33	0.00	25.00	

**Table 3:** Arbovirus similarity index according to Jaccard’s index among Neotropical countries.

Country	Argentina	Brazil	Colombia	Costa Rica	Guatemala	French Guiana	Mexico	Panama	Paraguay	Uruguay	Venezuela
**Argentina**											
**Brazil**	29.03										
**Colombia**	22.22	9.09									
**Costa Rica**	31.25	11.76	22.22								
**Guatemala**	14.29	13.79	23.08	25.00							
**French Guiana**	23.08	13.79	14.29	15.38	11.11						
**Mexico**	26.32	17.65	22.22	37.50	41.67	13.33					
**Panama**	33.33	15.15	66.67	27.78	33.33	14.29	38.89				
**Paraguay**	18.18	3.45	8.33	0.00	0.00	16.67	0.00	18.18			
**Uruguay**	18.18	3.45	7.69	20.00	16.67	16.67	16.67	16.67	0.00		
**Venezuela**	21.43	9.68	6.25	14.29	22.22	0.00	20.00	20.00	0.00	14.29	

**Table 4:** Similarity index according to Sorensen’s index among mammalian orders in the Neotropics.

Orders	Artiodactyla	Carnívora	Chiroptera	Didelphimorphia	Lagomorpha	Perissodactyla	Pilosa	Non-Human Primates	Rodentia
**Artiodactyla**									
**Carnívora**	0.00								
**Chiroptera**	30.00	20.00							
**Didelphimorphia**	22.22	25.00	61.54						
**Lagomorpha**	0.00	0.00	0.00	0.00					
**Perissodactyla**	34.48	0.00	32.00	26.09	0.00				
**Pilosa**	34.78	0.00	42.11	35.29	15.38	28.57			
**Non-human primates**	18.75	9.09	35.71	23.08	0.00	43.24	45.16		
**Rodentia**	19.35	19.05	51.85	32.00	9.52	33.33	46.67	46.15	

**Table 5:** Similarity index according to Jaccard’s index among mammal orders in the Neotropics.

Orders	Artiodactyla	Carnívora	Chiroptera	Didelphimorphia	Lagomorpha	Perissodactyla	Pilosa	Non-Human Primates	Rodentia
**Artiodactyla**									
**Carnívora**	0.00								
**Chiroptera**	17.65	11.11							
**Didelphimorphia**	22.22	14.29	44.44						
**Lagomorpha**	0.00	0.00	0.00	0.00					
**Perissodactyla**	20.83	0.00	19.05	15.00	0.00				
**Pilosa**	21.05	0.00	26.67	21.43	8.33	16.67			
**Non-human primates**	10.34	4.76	21.74	13.04	0.00	27.59	29.17		
**Rodentia**	10.71	10.53	35.00	19.05	5.00	20.00	30.43	30.00	

**Table 6:** Mammals positive to arbovirus in Neotropical countries and occurrence according to habitat.

	Positives Mammals to Arbovirus	Negatives Mammals to Arbovirus	Total
Mammals in deforested fronts	1312	3268	4580
Mammals in vegetation cover	1549	5634	7183
Total	2861	8902	11,763

## Appendix A

**Table A1:** Mammalian orders with arbovirus-positive records by habitat and environment.

Mammalian Host	Environment	Country	Arbovirus	Habitat	References
Artiodactyla	Domestic	Argentina Brazil Mexico	Apeu virus (APEUV) Guaroa virus (GROV) Venezuelan equine encephalitis virus (VEEV) Maguari virus (MAGV) Murutucu virus (MURV) Oriboca virus (ORIV) Oropouche virus (OROV) Tacaiuma virus (TCMV) West Nile virus (WNV) Xingu virus (XINV)	Primary forest Populated zone Not specified	[98,99,104,129]
	Wild	French Guiana Mexico	Saint Louis encephalitis virus (SLEV) Jamestown Canyon virus (JCV)	Primary fores Not specified	[105,114]
Carnívora	Wild	French Guiana Mexico	Dengue virus (DENV) Cache Valley virus (CVV)	Primary forest Not specified	[105,114]
Chiroptera	Wild	Brazil Colombia Costa Rica Guatemala French Guiana Mexico	Dengue virus (DENV) Eastern equine encephalitis virus (EEEV) Venezuelan Equine Encephalitis virus (VEEV) Saint Louis encephalitis virus (SLEV) Vesicular stomatitis, New Jersey virus (VSNJV) Vesicular stomatitis, Indiana (VSIV) Mojui dos Campos virus (MDCV) West Nile virus (WNV)	Primary forest Secondary forest Intervened area Not specified Populated zone	[108,109,113,116,117,118,120,133,138,281]
Didelphimorphia	Wild	Brazil Colombia French Guiana Panama Venezuela	Bunyamwera virus (BUNV) Catu virus (CATUV) Dengue virus (DENV) Venezuelan equine encephalitis virus (VEEV) Saint Louis encephalitis virus (SLEV) Vesicular stomatitis, Indiana virus (VSIV)	Primary forest Secondary forest Not specified Intervened area	[67,101,106,113,114,132]
Lagomorpha	Wild	Mexico Panama	Melao virus (MELV) Rio Grande virus (RGV)	Not specified	[105,106]
Perissodactyla	Domestic	Argentina Brazil Mexico Panama Uruguay Venezuela	ALPHA: alphavirus undetermined Bunyamwera virus (BUNV) Bussuquara virus (BSQV) Eastern equine encephalitis virus (EEEV) Weastern equine encephalitis virus (WEEV) Venezuelan equine encephalitis virus (VEEV) Saint Louis encephalitis virus (SLEV) Fort Sherman virus (FSV) Icoaraci virus (ICOV) Itaqui virus (ITQV) Madrid virus (MADV) Maguari virus (MAGV) Mayaro virus (MAYV) Rocio virus (ROCV) Tacaiuma virus (TCMV) West Nile virus (WNV) Xingu virus (XINV)	Secondary forest Not specified Populated zone	[98,99,100,104,119,122,123,125,126,129,130,131,135]
Pilosa	Wild	Brazil Costa Rica French Guiana	Changuinola virus (CGLV) Venezuelan equine encephalitis virus (VEEV) Saint Louis encephalitis virus (SLEV) Vesicular stomatitis, Indiana virus (VSIV) Yellow fever virus (YFV) Ilheus virus (ILHV) Punta Toro virus (PTV) Rio Grande virus (RGV) Tacaiuma virus (TCMV) West Nile virus (WNV) Zika virus (ZIKV)	Primary forest Secondary forest	[102,107,114,124]
Non-human primates	Domestic or Zoological settings	Brazil Costa Rica	Dengue virus (DENV) Yellow fever virus (YFV) Flavavirus indeterminado Fort Sherman virus (FSV) Oropouche virus (OROV)	Captive Populated zone	[110,111,134,136]
	Wild	Argentina Brazil Costa Rica French Guiana Panama Paraguay Venezuela	Bussuquara virus (BSQV) Cacipacore virus (CPCV) Caraparu virus (CARV) Changuinola virus (CGLV) Dengue virus (DENV) Eastern equine encephalitis virus (EEEV) Saint Louis encephalitis virus (SLEV) Vesicular stomatitis, Indiana virus (VSIV) Yellow fever virus (YFV) Icoaraci virus (ICOV) Ilheus virus (ILHV) Mayaro virus (MAYV) Oropouche virus (OROV) Rocio virus (ROCV) UNA virus (UNAV) Utinga virus (UTIV) West Nile virus (WNV) Zika virus (ZIKV)	Primary forest Secondary forest Intervened area Populated zone	[102,103,106,110,111,112,114,115,124,127,128,131,137,139,140,141]
Rodentia	Wild	Brazil Colombia French Guiana Mexico Panama Venezuela	Cache Valley virus (CVV) Changuinola virus (CGLV) Dengue virus (DENV) Eastern equine encephalitis virus (EEEV) Weastern equine encephalitis virus (WEEV)Venezuelan equine encephalitis virus (VEEV) Saint Louis encephalitis virus SLEV) Vesicular stomatitis, New Jersey virus (VSNJV) Vesicular stomatitis, Indiana virus (VSIV) Yellow fever virus (YFV) Gamboa virus (GAMV) Guama virus (GMAV) Ilheus virus (ILHV) Madrid virus (MADV) Melao virus (MELV) Ossa virus (OSSAV) UNA virus (UNAV) West Nile virus (WNV)	Primary forest Secondary forest Captive Not specified Intervened area	[67,101,105,106,113,114,121,132,196]

## Appendix B

**Table A2:** Representation of arbovirus-positive and -negative records in every type of area.

#	Title	Author	Prevalence	Positive	Negative	Type of Area
1	Arbovirus serosurvey Orthobunyavirus, Flavivirus, and Alphavirus) in a draft horse population from Santa Fe, Argentina 2013–2016)	Albrieu-Llinás 2021 [123]	TOTAL 222 = INFECTED prevalence FSV = 76.8%, SLEV = 59.6%, WNV = 27.5%, RNV = 7.4%	FSV: 170 SLEV: 132 WNV: 61 RNV: 16	FSV: 52 SLEV: 90 WNV: 161 RNV: 206	Deforested fronts
2	Prevalence of Flavivirus antibodies in Alouatta caraya primate autochthonous of Argentina	Contigiani et al., 2000 [127]	TOTAL 105 = INFECTED prevalence HI 35.23%), NT 32.38%)	37 34	68 71	Deforested fronts
3	Serological survey on arbovirus detected in animals in the province of Tucuman, Argentina	De Ruiz Holgado et al., 1967 [129]	Total 75 = infected 58	58	17	Deforested fronts
4	Infection by UNA virus Alphavirus; Togaviridae) and risk factor analysis in black howler monkeys Alouatta caraya) from Paraguay and Argentina	Díaz et al., 2007 [137]	TOTAL 90 = INFECTED 67 46 UNAV y 21 UNAV y el MAYV)	67	23	Deforested fronts
5	First isolation of Bunyamwera virus Bunyaviridae family) from horses with neurological disease and an abortion in Argentina	Tauro et al., 2015 [100]	2 HORSES = 2 INFECTED	2	0	Deforested fronts
6	Neutralizing antibodies for orthobunyaviruses in Pantanal, Brazil	Pauvolid-Corrêa et al., 2017 [99]	TOTAL 607 = 373 equidos + 126 bovinos INFECTED TOTAL 499	499	108	Deforested fronts
7	Identification of animal hosts of Fort Sherman virus, a New World zoonotic orthobunyavirus	de Oliveira Filho et al., 2020 [135]	TOTAL 192 = 2 INFECTED	2	190	Deforested fronts
8	Yellow fever surveillance challenge: Investigation of a marmoset non-autochthonous case	Fernandes et al., 2020 [134]	TOTAL 1 = 1 INFECTED	1	0	Vegetation cover
9	Identification of the encephalitis equine virus, Parana, Brazil	Fernández et al., 2000 [119]	TOTAL 22 = INFECTED 12	12	10	Deforested fronts
10	Detection of antibodies to Oropouche virus in non-human primates in Goiânia City, Goiás	Gibrail et al., 2016 [136]	TOTAL 50 = 2 INFECTED	2	48	Vegetation cover
11	Neutralising antibodies for Mayaro virus in Pantanal, Brazil	Pauvolid-Corrêa et al., 2015 [98]	TOTAL 985 = 239 INFECTED	239	746	Vegetation cover
12	A Saint Louis encephalitis and Rocio virus serosurvey in Brazilian horses	Silva et al., 2014 [130]	TOTAL 753 = INFECTED 415	415	338	Vegetation cover
13	Serological evidence for Saint Louis encephalitis virus in free-ranging New World monkeys and horses within the upper Paraná River basin region, Southern Brazil	Svoboda et al., 2014 [131]	TOTAL 133 = 30 INFECTED	30	103	Vegetation cover
14	Ultrastructural, antigenic and physicochemical characterization of the Mojuí dos Campos Bunyavirus) isolated from bat in the Brazilian Amazon region	Wanzeller et al., 2002 [138]	TOTAL 1 = INFECTED 1	1	0	Deforested fronts
15	A survey to assess potential human disease hazards along proposed sea level canal routes in Panamá and Colombia. V. Arbovirus infection in non-human vertebrates	Srihonges et al., 1974 [106]	TOTAL 2214 = INFECTED 28	28	2186	Vegetation cover
16	Serosurvey of selected arboviral pathogens in free-ranging, two-toed sloths (*Choloepus hoffmanni*) and three-toed sloths (*Bradypus variegatus*) in Costa Rica, 2005-07	Medlin et al., 2016 [107]	TOTAL 109 = 87 INFECTED	87	22	Vegetation cover
17	Serologic survey of neotropical bats in Guatemala for virus antibodies	Ubico et al., 1995 [120]	TOTAL 332 = INFECTED 87	87	245	Vegetation cover
18	Dengue infection in neotropical forest mammals	de Thoisy et al., 2009 [113]	TOTAL 616 = INFECTED 92	92	524	Vegetation cover
19	Health evaluation of translocated free-ranging primates in French Guiana	de Thoisy et al., 2001 [112]	TOTAL 141 = INFECTED	Dengue virus II 20/141, Yellow fever virus 47/141, Saint Louis virus 16/141, Mayaro virus 76/141 Average 40	Dengue virus II 121/141, Yellow fever virus 94/141, Saint Louis virus 125/141, Mayaro virus 65/141 Average 101	Vegetation cover
20	Serologic survey for selected arboviruses and other potential pathogens in wildlife from Mexico	Aguirre et al., 1992 [105]	TOTAL 80 = 1 INFECTED	1	79	Deforested fronts
21	Serologic survey of domestic animals for zoonotic arbovirus infections in the Lacandón Forest region of Chiapas, Mexico	Ulloa et al., 2003 [104]	TOTA 72 = INFECTED 54	54	18	Vegetation cover
22	Human and Equine Infection with Alphaviruses and Flaviviruses in Panamá during 2010: A Cross-Sectional Study of Household Contacts during an Encephalitis Outbreak	Carrera et al., 2018 [126]	TOTAL 194 = INFECTED 126	126	68	Vegetation cover
23	Seroprevalence of St. Louis encephalitis virus and West Nile virus Flavivirus, Flaviviridae) in horses, Uruguay	Burgueño et al., 2013 [125]	TOTAL 425 = INFCTED 205	205	220	Deforested fronts
24	Ecological studies of enzootic Venezuelan equine encephalitis in north-central Venezuela, 1997–1998	Salas et al., 2001 [101]	TOTAL 543 = INFECTED 30	30	513	Deforested fronts
25	Studies of arboviruses in Southwestern Venezuela: I. Isolations of Venezuelan and Eastern Equine Encephalitis viruses from sentinel hamsters in the Catatumbo region	Walder et al., 1976 [121]	TOTAL 95 = INFECTED 30	30	65	Deforested fronts
26	Arbovirus studies in the Guajira region of Venezuela: activities of eastern equine encephalitis and Venezuelan equine encephalitis viruses during an interepizootic period	Walder et al., 1984 [122]	TOTAL 64 = INFECTED 60	60	4	Deforested fronts
27	Detection of dengue virus in bat flies Diptera: Streblidae) of common vampire bats, Desmodus rotundus, in Progreso, Hidalgo, Mexico	Abundes-Gallegos et al., 2018 [108]	TOTAL 16 = INFECTED 8	8	8	Vegetation cover
28	Detection of antibodies against Icoaraci, Ilhéus, and Saint Louis Encephalitis arboviruses during yellow fever monitoring surveillance in non-human primates Alouatta caraya) in southern Brazil	Almeida et al., 2019 [124]	TOTAL 26 = INFECTED 5	5	21	Deforested fronts
29	Contrasting sylvatic foci of Venezuelan equine encephalitis virus in northern South America	Barrera et al., 2002 [132]	TOTAL 546 = INFECTED 20	20	526	Deforested fronts
30	Study of Arboviruses in Philander opossum, Didelphis marsupialis and Nectomys rattus captured from forest fragments in the municipality of Belém, Pará, Brazil	Bernal et al., 2021 [67]	TOTAL 14 = INFECTED 8	8	6	Deforested fronts
31	Dengue virus infection in neotropical forest mammals: incidental hosts or potential reservoirs?	Lavergne et al., 2009 [281]	TOTAL 464 = INFECTED 92	92	372	Vegetation cover
32	Two Cases of Natural Infection of Dengue-2 Virus in Bats in the Colombian Caribbean	Calderón et al., 2021 [109]	TOTAL 286 = INFECTED 2	2	284	Deforested fronts
33	Surveillance of arboviruses in primates and sloths in the Atlantic Forest, Bahia, Brazil	Catenacci et al., 2018 [102]	TOTAL 139 = INFECTED 25	25	114	Vegetation cover
34	Flaviviruses infections in neotropical primates suggest long-term circulation of Saint Louis Encephalitis and Dengue virus spillback in socioeconomic regions with high numbers of Dengue human cases in Costa Rica	Chaves et al., 2020 [110]	TOTAL 86 = INFECTED 35	35	51	Vegetation cover
35	Serosurvey of Nonhuman Primates in Costa Rica at the Human–Wildlife Interface Reveals High Exposure to Flaviviruses	Chaves et al., 2021 [111]	TOTAL 86 = INFECTED 38	38	48	Vegetation cover
36	Immunity to yellow fever, Oropouche and Saint Louis viruses in a wild howler monkey	de Almeida et al., 2016 [128]	TOTAL 1 = INFECTED 1	1	0	Deforested fronts
37	Detection of a novel African-lineage-like Zika virus naturally infecting free-living neotropical primates in Southern Brazil	de Almeida et al., 2019 [140]	TOAL 50 = INFECTED 9	9	41	Vegetation cover
38	Wild terrestrial rainforest mammals as potential reservoirs for flaviviruses yellow fever, dengue 2 and St Louis encephalitis viruses) in French Guiana	De Thoisy, B Dussart, Philippe Kazanji, M. 2004 [114]	TOTAL 574= INFECTED 50	50	524	Vegetation cover
39	Zika Virus in peridomestic neotropical primates, Northeast Brazil	Favoretto, Silvana et al., 2019 [141]	TOTAL 132 = INFECTED 9	9	123	Deforested fronts
40	Detection of antibodies against flavivirus over time in wild non-human primates from the lowlands of Costa Rica	Dolz, Gaby et al., 2019 [115]	TOTAL 209 = INFECTED 53	53	156	Deforested fronts
41	Serologic evidence of flavivirus infection in bats in the Yucatan Peninsula of Mexico	Machain-Williams, Carlos et al., 2013 [116]	TOTAL 140 = infected 26	26	114	Deforested fronts
42	Neotropical bats that co-habit with humans function as dead-end hosts for dengue virus	Vicente-Santos, Amanda et al., 2017 [118]	TOTAL 318 = INFECTED 28	28	290	Deforested fronts
43	Dengue virus in bats from southeastern Mexico	Sotomayor-Bonilla, Jesús et al., 2014 [117]	TOTAL 146 = INFECTED 79	79	67	Vegetation cover
44	Isolation of Madre de Dios Virus Orthobunyavirus; Bunyaviridae), an Oropouche virus species reassortant, from a monkey in Venezuela	Navarro et al., 2016 [139]	TOTAL 2 = INFECTED 1	1	1	Deforested fronts
45	Eco-epidemiology of the Venezuelan equine encephalitis virus in bats of Cordoba and Sucre, Colombia	Guzmán, Camilo et al., 2019 [133]	TOTAL 286 = INFECTED 2	2	284	Deforested fronts
46	Detection of the mosquito-borne flaviviruses, West Nile, dengue, Saint Louis encephalitis, Ilheus, Bussuquara, and yellow fever in free-ranging black howlers *Alouatta caraya*) of northeastern Argentina	Morales et al., 2017 [103]	TOTAL 108 = INFECTED 70	70	38	Deforested fronts
